# Radiation Mapping: A Gaussian Multi-Kernel Weighting Method for Source Investigation in Disaster Scenarios

**DOI:** 10.3390/s25154736

**Published:** 2025-07-31

**Authors:** Songbai Zhang, Qi Liu, Jie Chen, Yujin Cao, Guoqing Wang

**Affiliations:** 1 School of Physics and Electronic Engineering, Sichuan University of Science & Engineering, Zigong 643000, China; 2 Intelligent Perception and Control Key Laboratory of Sichuan Province, Sichuan University of Science & Engineering, Zigong 643000, China; 323085404202@stu.suse.edu.cn (J.C.); 323085404301@stu.suse.edu.cn (Y.C.); 323085404223@stu.suse.edu.cn (G.W.); 3 School of Automation and Information Engineering, Sichuan University of Science & Engineering, Zigong 643000, China

**Keywords:** radiation mapping, multi-kernel regression, GPR, source localization, SLAM

## Abstract

Structural collapses caused by accidents or disasters could create unexpected radiation shielding, resulting in sharp gradients within the radiation field. Traditional radiation mapping methods often fail to accurately capture these complex variations, making the rapid and precise localization of radiation sources a significant challenge in emergency response scenarios. To address this issue, based on standard Gaussian process regression (GPR) models that primarily utilize a single Gaussian kernel to reflect the inverse-square law in free space, a novel multi-kernel Gaussian process regression (MK-GPR) model is proposed for high-fidelity radiation mapping in environments with physical obstructions. MK-GPR integrates two additional kernel functions with adaptive weighting: one models the attenuation characteristics of intervening materials, and the other captures the energy-dependent penetration behavior of radiation. To validate the model, gamma-ray distributions in complex, shielded environments were simulated using GEometry ANd Tracking 4 (Geant4). Compared with conventional methods, including linear interpolation, nearest-neighbor interpolation, and standard GPR, MK-GPR demonstrated substantial improvements in key evaluation metrics, such as *MSE*, *RMSE*, and *MAE*. Notably, the coefficient of determination (*R*^2^) increased to 0.937. For practical deployment, the optimized MK-GPR model was deployed to an RK-3588 edge computing platform and integrated into a mobile robot equipped with a NaI(Tl) detector. Field experiments confirmed the system’s ability to accurately map radiation fields and localize gamma sources. When combined with SLAM, the system achieved localization errors of 10 cm for single sources and 15 cm for dual sources. These results highlight the potential of the proposed approach as an effective and deployable solution for radiation source investigation in post-disaster environments.

## 1. Introduction

While radiation characterization via traditional manual surveys remains fundamental to decommissioning, safeguarding, and nonproliferation efforts in both routine and emergency scenarios, it inherently suffers from significant limitations, including elevated occupational radiation exposure risks and inefficiencies in data acquisition [1,2].

Mobile robotic systems equipped with SLAM technology offer a promising solution for autonomous radiation field mapping in high-risk environments [3,4]. This approach enables high-resolution spatial mapping of radiation distribution, enhances the identification of potential radiological hazards, and provides essential data to analyze the following emergency measures and decommissioning means of nuclear facilities. As a result, radiation mapping technology has become increasingly vital for ensuring nuclear safety and security [5].

With the rapid advancement of robotic technologies, autonomous mobile platforms equipped with radiation detection capabilities have emerged as a leading solution for automated surveys, effectively substituting manual operations in hazardous settings [6,7,8,9]. These systems typically integrate environmental perception modules with radiation sensing capabilities, enabling autonomous navigation and real-time radiation distribution reconstruction. Such integration has facilitated novel methodologies for radiation mapping, offering significant advantages over traditional manual survey techniques in terms of safety and operational efficiency [10,11].

Autonomous robotic systems equipped with radiation detection and SLAM technology offer significant potential for nuclear safety, post-accident radiation assessment, and decommissioning of nuclear facilities. For instance, in scenarios like the Fukushima Daiichi nuclear disaster, where complex indoor environments and radioactive debris pose challenges, such systems can map radiation fields and localize sources, reducing human exposure risks [12,13]. These capabilities are critical for mapping radioactive debris in damaged nuclear power plants and supporting emergency source localization in contaminated indoor settings, enhancing operational safety and efficiency.

Recent literature indicates that radiation mapping methodology broadly follows two trajectories: multi-sensor data fusion and algorithmic processing of radiation distribution reconstruction. In the domain of data fusion, the accurate integration of radiation intensity measurements with geometric and spatial information remains a core requirement for constructing high-resolution radiation distribution maps [14]. Significant progress has been achieved through the development of SLAM techniques and Robot Operating System (ROS) frameworks, which provide a foundation for integrating heterogeneous sensor data streams [15,16]. For instance, Besada-Portas et al. [17] proposed a time delay estimation (TDE) algorithm to temporally align data from positioning sensors, radiation detectors, and robotic systems, thereby improving multi-source data synchronization accuracy.

In parallel, the combination of multimodal data fusion with adaptive interpolation algorithms has advanced the accuracy and the robustness of radiation distribution reconstructions in complex environments [18]. Furthermore, the deployment of unmanned aerial vehicles (UAV) has gained traction as an effective means of conducting radiation surveys, particularly in large-scale or obstacle-rich outdoor areas. Recent studies have proposed UAV-based frameworks for radiation scouting, real-time monitoring, and automated inspection, incorporating 3D mapping, radiological heatmap generation, and hotspot localization through coordinated multi-drone operations. These systems have demonstrated high efficiency, scalability, and operational resilience in field scenarios [19,20].

At the algorithmic level, research primarily has concentrated on spatial interpolation of radiation measurements to generate continuous radiation distribution maps [21]. Early studies explored numerical approximation methods and classical Bayesian inference models [22,23]. However, these approaches exhibit limited effectiveness in scenarios involving complex and heterogeneous radiation distributions. For example, the inverse distance weighted (IDW) interpolation method introduced by Kikawa et al. [24] achieved sub-meter accuracy in sparse datasets, while the high sensitivity to measurement noise often results in artifacts and distortions in the reconstructed radiation maps.

To improve robustness under irregular sampling conditions, West et al. [25] pioneered the application of Gaussian process regression (GPR) for radiation distribution interpolation. GPR has demonstrated superior adaptability in handling noisy background, irregular measurement intervals, and low-count radiation statistics, establishing itself as a reference methodology in this domain. Subsequent enhancements incorporated Matérn covariance functions (Matérn 3/2 kernel) and radial basis functions (RBF kernel), improving prediction accuracy and reducing localization errors of radiation source to within 0.5 m without obstacles or shielding conditions [26,27]. Based on this foundation, Jung et al. [28] proposed an inverse-square law-derived kernel (InvSq Kernel) for use within the GRP framework, enabling 3D radiation distribution visualization through the spatial projection of predicted intensity results.

Despite advancements in radiation field mapping, conventional interpolation methods face significant challenges in environments obstructed by accident-induced collapse, such as indoor settings with concrete or steel debris. The inverse-square kernel, for instance, exhibits reduced accuracy due to γ-ray shielding effects. These limitations stem from three primary factors:

(1) Sparse or incomplete sampling: Obstacle-constrained navigation paths result in a non-uniform spatial distribution of measurement points and the potential omission of critical survey locations.
(2) Radiation field distortion: Variability in obstacle types and material thicknesses introduces significant radiation attenuation effects, leading to distortions in the spatial distribution of the original radiation field.
(3) Modeling methodology constraints: Existing interpolation algorithms lack robust integration mechanisms for incorporating material-specific attenuation coefficients, resulting in systematic discrepancies between reconstructed radiation maps and actual environmental measurements.

The challenges of sparse sampling, radiation field distortion, and inadequate modeling of material-specific attenuation coefficients hinder accurate radiation field mapping and practical deployment in nuclear safety applications. Therefore, robust methods are needed to address these limitations in obstructed environments.

This study introduces an MK-GPR framework designed for obstructed environments. By combining a free-space inverse-square kernel with a material-specific decay kernel and incorporating adaptive weight assignment and SLAM for grid-based mapping, this method achieves robust and accurate radiation field reconstruction, even in data-sparse, complex scenarios, supporting nuclear monitoring and emergency response applications.

## 2. Multi-Kernel Weighted Gaussian Process for Obstacle Attenuation Modeling

### 2.1. Gaussian Theory Framework

GPR employs a single covariance function to model the similarity between data points. As a Bayesian non-parametric approach, GPR defines a prior over function space (initial probabilistic assumptions) in the form of a Gaussian process and updates it through posterior inference based on observed data. Formally, a Gaussian process generalizes multivariate Gaussian distributions to infinite-dimensional function spaces, fully specified by a mean function m(x) and a covariance function $K(x,x^{\prime })$, as defined in the following Equation (1):

(1) $$f(x)\sim GP(m(x),K(x,x^{\prime }))$$

Here, f(x) denotes a random function modeled by a Gaussian process. The joint distribution of its evaluation at any finite set of input points is multivariate Gaussian, determined by the chosen kernel function. Typically, the mean function m(x) is assumed to be zero for simplicity, while the covariance function $K(x,x^{\prime })$—also known as the kernel—quantifies similarity between any pair of input points, x and $x^{\prime }$. The common kernel choices include the squared exponential (radial basis function, RBF) kernel and polynomial kernels. The nature of the inputs is application-dependent; in this context, they correspond to spatial positions within the environment.

For radiation fields with obstacles, the spatial distribution of radiation intensity is highly irregular. This complexity arises from the nonlinear interplay between radiation attenuation over distance and occlusion effects introduced by obstacles. A single-kernel function might be insufficient to model such intricate spatial dependencies. To address this limitation, the MK-GPR is proposed. This approach extends the Bayesian framework by assigning adaptive weights $\omega_{i}$ to multiple kernel functions and composes a compound covariance function, as shown in the following Equation (2). This formulation enhances the model’s expressiveness and adaptability to the heterogeneous characteristics of complex radiation distributions.

(2) $$K(x,x^{\prime })=\sum_{i=1}^{j}\omega_{i}K_{i}\left(x,x^{\prime }\right)$$

where $\omega_{i}$ denotes the weight coefficient for each kernel, subject to the constraint $\sum_{i=1}^{j}\omega_{i}=1$. They are typically determined through the optimization approach using marginal likelihood maximization, enabling the model to simulate the most relevant similarity measures for a given spatial configuration.

By integrating multiple kernels, the MK-GPR framework could offer a more flexible and expressive prior over function, especially for radiation distribution patterns with localized discontinuities, anisotropic decay, or abrupt occlusion effects due to the obstacles. In this study, the basic kernel functions are composed by physical principles, such as the inverse-square law governing radiation intensity attenuation with respect to distance, further accounting for the heterogeneous attenuation characteristic introduced by different obstacle materials—aluminum or iron. Customized attenuation kernels are designed for each material type. These different kernels explicitly model the material-specific absorption and scattering effects on radiation transportation. By employing the MK-GPR, the model integrates the physically informed kernels into a unified covariance structure to simulate the radiation fluctuation with spatial distance and obstacles. Thereby, the model substantially improves the fidelity of radiation reconstruction and enhances the precision of radiation localization in complex environments.

### 2.2. Kernel Functions of Common Obstacles in Radiation Fields

The spatial distribution of radiation intensity in a free space follows the classic inverse-square law. Specifically, the radiation intensity $I_{\left(r\right)}$ at a distance r from the source is inversely proportional to the square of the distance, as the following Equation (3) shows:

(3) $$I_{\left(r\right)}=\frac{I_{0}}{r^{2}}$$

where $I_{0}$ denotes the initial radiation intensity at the source, and r represents the Euclidean distance between the source and the observation point.

While this equation characterizes the theoretical radiation distribution without any obstacles, it does not define a covariance structure required for assessing similarity between different spatial locations. To enable its integration with a Gaussian process framework, the inverse-square formulation should be transformed into a valid Gaussian kernel function, as illustrated in Equation (4):

(4) $$K_{r}(r,r^{\prime })=\frac{\sigma^{2}}{{\left\|r-r^{\prime }\right\|}^{2}+\varepsilon }$$

In this formulation, $\sigma^{2}$ represents the radiation counts variance, which determines the overall scale of the kernel function. The term ${\left\|r-r^{\prime }\right\|}^{2}$ denotes the squared Euclidean distance between two points, while ε is a small regularization constant (typically set to a very small positive value, such as ε=0.05) introduced to reduce numerical singularities. This kernel function is well suited for modeling open environments without obstacles; however, it presents significant limitations when applied to complex environments rich with obstacles.

In such cases, a new attenuation-aware kernel function should be constructed to account for the influence of shielding. Leveraging the known attenuation properties of gamma radiation in various media, an alternative kernel function could be formulated based on an exponential attenuation model. This approach enables quantitative modeling of radiation decay due to shielding by obstacles. The exponential attenuation model is defined as follows:

(5) $$I(d)=I_{0}e^{-\mu d}$$

where $I_{0}$ is the initial radiation intensity,μ is the linear attenuation coefficient of the medium, and d is the effective path length through the medium. The kernel function incorporates the attenuation properties of gamma ray, as shown in the following Equation (6):

(6) $$K(d,d^{\prime })=\sigma^{2}exp(-\mu \left\|d-d^{\prime }\right\|)$$

This kernel function measures spatial correlation based on the accumulated differences by varying material thicknesses, where $\left\|d-d^{\prime }\right\|$ denotes the difference in effective path length due to heterogeneous media. Compared to the traditional squared exponential (RBF) kernel, the exponential kernel exhibits great sensitivity to abrupt variations in radiation intensity, making it particularly effective for simulating steep attenuation effects in obstacle-rich areas.

In practical applications, attenuation kernel functions must be tailored to the specific material present in the paths of gamma rays. The linear attenuation coefficients for different materials at various gamma-ray energies could be obtained from the NIST XCOM database [29]. For example, if the gamma ray penetrates aluminum or iron media to the detector from two radioactive sources, ^137^Cs and ^60^Co, the gamma from ^137^Cs (662 keV) and ^60^Co (1173 keV/1332 keV) experience exponential attenuation. The corresponding attenuation coefficients could be extracted from this database. In the case of ^60^Co, whose gamma spectrum features two prominent energy peaks, an energy-weighted fusion of attenuation coefficients is performed to ensure the kernel function accurately reflects the composite spectral behavior. The attenuation coefficients (units: $m^{\;-\;1}$) are summarized in Table 1.

These coefficients are then integrated into a unified material-aware attenuation kernel function defined as follows:

(7) $$K_{E}^{m}(d_{m},d_{m}^{\prime })=\sigma^{2}exp(-\mu_{E}^{m}\left\|d_{m}-d_{m}^{\prime }\right\|)$$

Here, $\mu_{E}^{m}$ denotes the linear attenuation coefficient as a function of photon energy (E) across various materials (m), and $\left\|d_{m}-d_{m}^{\prime }\right\|$ represents the effective path lengths through materials. This kernel captures the influence of heterogeneous shielding media on radiation correlation.

To model radiation fields in environments with obstacles, we construct a composite kernel function by combining the free-space kernel and multiple material-specific attenuation kernels through a log-domain weighted product formulation, as shown in Formula (8):

(8) $$K=\exp\left(\omega_{1}\log K_{r}(r,r^{\prime })+\sum_{m=2}^{5}\omega_{m}\log K_{E}^{m}(d_{m},d_{m}^{\prime })\right)$$

This structure reflects the layered attenuation process that gamma rays undergo as they propagate through both free space and obstructing materials. The logarithmic weighting ensures smoother optimization behavior and maintains the physical interpretability of kernel contributions.

Within obstructed regions, the weighting coefficients $\sum_{m=1}^{5}\omega_{m}=1$ are assigned based on the spatial distribution and types of materials present, modulating the influence of each kernel component accordingly. In free-space regions, where no obstacles are encountered, the kernel simplifies to the form governed solely by $K_{r}(r,r^{\prime })$, which models the inverse-square law of radiation decay.

The weight coefficients $\omega_{m}$ and attenuation coefficients $\mu_{E}^{m}$ are determined based on prior information. The weights are pre-assigned using spatial semantic or geometric information, such as point cloud data from LiDAR mapping. The workflow involves: (a) capturing environmental geometry via LiDAR point clouds, (b) performing spatial semantic analysis to identify material types and their attenuation coefficients, (c) initializing weights based on geometric and material information, and (d) optimizing weights $\omega_{m}$ for the inverse-square law and material-specific kernels by maximizing the marginal log-likelihood using gradient descent. The counts variance $\sigma^{2}$ is optimized by maximizing the marginal log-likelihood of the observed radiation counts. Due to the low dimensionality of the parameter space, gradient-based optimization is more efficient, with the loss function typically converging to a global optimum within approximately 80 iterations.

### 2.3. Comparison of Different Interpolation Methods in Obstacle Environments

To evaluate the interpolation performance of different methods for radiation distribution reconstruction in the case of obstacles, a ^137^Cs gamma radiation distribution was simulated by GEANT4. The source located at the origin of a Cartesian coordinate system (0, 0) and a circular obstacle with a radius of 20 cm were introduced at coordinates (30, 30). This obstacle resulted in approximately 12.56% of the spatial domain being occluded, leading to observation data loss.

**Figure 1:**
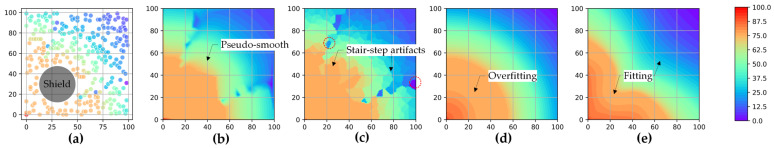
Comparison of four interpolation techniques: (**a**) sampling points with obstacle region indicated, (**b**) linear interpolation, (**c**) nearest-neighbor interpolation, (**d**) Gaussian single-kernel interpolation, and (**e**) Gaussian multi-kernel interpolation. Radiation counts are normalized between 0 and 100, visualized using the color scale shown in the legend, where the color gradient transitions from blue to yellow to red, representing increasing radiation counts intensity.

In the experiment, a total of 450 non-uniformly distributed sampling points were randomly selected across the domain. The obstacle-affected area and the sampling points are illustrated in Figure 1a. Among them, 300 sampling points were used for interpolation, while the remaining 150 points were retained for validation and error analysis. Four interpolation methods were compared in this study: linear interpolation, nearest-neighbor interpolation, Gaussian single-kernel interpolation, and Gaussian multi-kernel weighted interpolation. The performance of each method is visualized in Figure 1.

Figure 1 presents the reconstructed distribution results using the four different interpolation methods, where the circular region in Figure 1a indicates the obstacle location. A summary of performance characteristics is analyzed, as shown below.

Linear interpolation (Figure 1b): Linear interpolation estimates unknown values by assuming a linear variation between neighboring data points. It ensures continuity of radiation values in densely sampled regions and offers high computational efficiency. However, it is highly sensitive to abrupt variations in the radiation distribution introduced by obstacles and leads to “pseudo-smoothing” effects near obstacle boundaries, where attenuation discontinuities are smoothed out unrealistically. Consequently, this method fails to accurately represent the attenuation behind obstacles, with poor accuracy in sparsely sampled regions.

Nearest-neighbor interpolation (Figure 1c): Nearest-neighbor interpolation assigns the value of the closest known point to the unknown location, which preserves data boundaries but leads to discontinuities and oversimplification. This method retains the local extrema of the original data and demonstrates strong robustness against sparse sampling and measurement noise. Nevertheless, it introduces step-like artifacts, leading to distorted spatial gradients and serrated boundary effects around the obstacle. These artifacts might reduce spatial resolution and increase localization errors. Moreover, due to the retention of local extrema, this method cannot effectively handle outliers (such as those marked by red circles in Figure 1c) under significant noise conditions.

Gaussian single-kernel interpolation (Figure 1d): Gaussian single-kernel interpolation, typically based on Gaussian process regression (GPR) with a fixed kernel, provides smooth and continuous estimation while modeling spatial correlations. In this work, a modified inverse square (InvSq) kernel derived from the inverse-square law [28] is employed to achieve a globally smooth fit of the radiation field, consistent with the attenuation pattern in free space without obstacles. However, it performs poorly in regions affected by obstacles, failing to reflect local discontinuities and gradient transitions. This results in overfitting in smooth areas and significantly elevated prediction variance near obstacle boundaries.

Gaussian multi-kernel weighted interpolation (Figure 1e): The MK-GPR combines multiple kernels, weighted based on spatial and material-specific characteristics, to simultaneously capture global trends and local variants. It effectively models sharp gradient transitions at obstacle boundaries while preserving global smoothness. Reduced overfitting: Compared with Gaussian single-kernel interpolation, the MK-GPR method suppressed overfitting near obstacle edges, maintaining smoothness in open spaces while accurately modeling sharp intensity gradients at boundaries. The result is a more faithful topological reconstruction of radiation distribution with improved dose prediction accuracy and generalization capability in complex environments.

To comprehensively compare the performances of different interpolation methods in obstacle environments, a multi-metric evaluation system was established, incorporating root mean square error (*RMSE*), mean square error (*MSE*), mean absolute error (*MAE*), and the coefficient of determination (*R*^2^), as shown in Table 2.

(1) Mean square error (*MSE*) reflects the average squared difference between predicted values $y_{i}$ and actual values ${\hat{y}}_{i}$, exhibiting high sensitivity to outliers:

(9) $$MSE=\frac{1}{n}\sum_{i=1}^{n}{\left(y_{i}-{\hat{y}}_{i}\right)}^{2}$$

(2) Root mean square error (*RMSE*) retains the same units as the original data and directly reflects the absolute magnitude of prediction errors:

(10) $$RMSE=\sqrt{MSE}$$

(3) Mean absolute error (*MAE*) measures the average magnitude of prediction errors and is more robust to outliers, compared to *MSE* or *RMSE*:

(11) $$MAE=\frac{1}{n}\sum_{i=1}^{n}\left|y_{i}-{\hat{y}}_{i}\right|$$

(4) Coefficient of determination (*R*^2^) quantifies the interpolation model’s ability to explain the spatial variability of the radiation distribution through variance decomposition:

(12) $$R^{2}=1-\frac{\sum_{i=1}^{n}\left|y_{i}-{\hat{y}}_{i}\right|}{\sum_{i=1}^{n}\left|y_{i}-\bar{y}\right|}$$

where $\bar{y}$ denotes the mean radiation intensity over the entire domain. An $R^{2}$ value approaching 1 indicates that the model effectively captures the nonlinear distortions in the radiation field caused by obstacles.

Although linear interpolation offers high computational efficiency ($R^{2}$ = 0.868), it exhibits the highest *RMSE* (8.65) and *MAE* (6.98) among all methods. This is mainly attributed to the pseudo-smoothing effect near the obstacle boundary, which is caused by systematic deviations and limits its ability to accurately capture abruptly change in radiation counts.

The nearest-neighbor interpolation achieves a relatively low *MAE* (5.58), indicating better local accuracy. However, its *RMSE* (8.42) remains high, reflecting significant outlier errors. These stem from step-like artifacts that distort local gradients (as shown in Figure 1c), causing abnormal over- or underestimations near obstacles (highlighted by red circles in Figure 1c). Consequently, its *MSE* (70.98) remains elevated, indicating high noise sensitivity.

Gaussian single-kernel interpolation improves $R^{2}$ to 0.879 by modeling the global attenuation behavior in obstacles. Nevertheless, its *RMSE* (8.28) is still 35.1% higher than that of the multi-kernel method, reflecting the limitations of a single kernel in simultaneously capturing geometric and material-specific attenuation effects of obstacles. The large gap between its *MAE* (6.22) and *MSE* (68.54) further indicates persistent overfitting in certain regions.

In contrast, Gaussian multi-kernel weighted interpolation achieves consistent error reduction across all metrics. It yields the lowest *MAE* (4.61), reflecting enhanced preservation of local extrema while also reducing *RMSE* (5.97) and *MSE* (35.63), effectively suppressing anomalies near the obstacle boundary. The $R^{2}$ value reaches 0.937, indicating that 93.7% of the spatial variation in the radiation distribution is accurately reconstructed. Notably, the interpolated attenuation zones behind obstacles (indicated by the arrows in Figure 1e) closely align with physical attenuation laws.

Overall, experimental results demonstrate that the Gaussian multi-kernel weighting method outperforms traditional approaches in complex obstacle environments. Compared to linear methods, it reduces *RMSE* and *MAE* by 31.0% and 33.9%, respectively, while increasing $R^{2}$ to 0.937. Moreover, it maintains continuity in the primary radiation field (*MSE* reduced by 52.4%) and accurately reconstructs the secondary scattered field behind obstacles (highlighted by the yellow contours in Figure 1e). By leveraging the multi-kernel mechanism, this model not only describes spatial radiation attenuation but also quantitatively characterizes the shielding effects of obstacles, providing a robust foundation for high-precision radiation mapping in obstructed environments.

To address the computational considerations of the proposed multi-kernel Gaussian process regression (MK-GPR) method, we evaluated its computational complexity relative to traditional interpolation methods. The MK-GPR method, due to its use of multiple kernels and adaptive weight optimization via the marginal likelihood maximization equation (2), incurs a higher computational load, with an approximate time complexity of O(*n*^3^) for *n* data points, compared to O(*n*) for linear interpolation and O(*n* log *n*) for nearest-neighbor interpolation. The algorithms were deployed on an RK3588 hardware platform running a 64-bit Ubuntu 22.04 operating system, equipped with an 8-core CPU (4 × Cortex-A76 + 4 × Cortex-A55), a 3-core NPU, 16 GB of RAM, and 256 GB of storage. In our experiments, MK-GPR required approximately 1.2 s to process 200 measurement points on the RK3588 platform, with a load average of 4.13. However, the increased computational cost was offset by a significant reduction in localization error (Table 2), with an *R*^2^ value reaching 0.937.

To investigate the impact of increased sampling on traditional methods, we conducted a simulation by doubling the sample size for linear interpolation (from 200 to 400 points). The results showed a marginal improvement in *RMSE* (from 8.65 to 8.12) but still fell short of MK-GPR’s performance (*RMSE* 5.97). This suggests that the superior accuracy of MK-GPR is not solely dependent on sample size but on its ability to model complex attenuation patterns.

In emergency scenarios, where total response time is critical, the trade-off between computational and measurement overheads must be considered. Compared to the increase in computational time, the improvement in radiation field inversion accuracy achieved by MK-GPR in obstacle-rich environments is significant. Although the MK-GPR method requires additional computational resources, its processing time is significantly shorter than the measurement time, making the trade-off of minimal computational time for a substantial improvement in reconstruction accuracy worthwhile.

## 3. Radiation Distribution Mapping Experiment

### 3.1. Experimental Setup for Radiation Mapping

**Figure 2:**
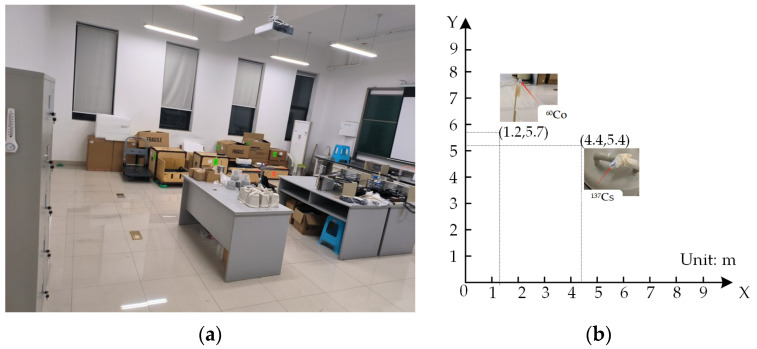
Experimental environment and radiation source placement. (**a**) Layout of the laboratory environment with obstacles. (**b**) Configuration of radiation source positions within the experimental domain.

The experiments were conducted in an enclosed laboratory space measuring 8 m × 8 m, with a Cartesian coordinate system established for spatial calibration (origin set at the southwest corner). To simulate a complex scene with obstacles, two representative materials—aluminum (density 2.7 g/cm^3^) and iron (density 7.87 g/cm^3^)—were arranged to form an asymmetric topological structure, introducing varying degrees of disruption to radiation distribution. Additionally, a pile of boxes near the window was arranged to mimic the collapse of indoor objects following an earthquake or explosion, simulating a post-disaster scenario with structural damage and debris accumulation. This setup enhanced the complexity of radiation field mapping by introducing unpredictable shielding and scattering effects, reflecting real-world disaster conditions. The experimental layout, encompassing obstacle placement and radiation source positions, is depicted in Figure 2.

The experiment was conducted under two configurations: a single-source setup (^37^Cs) and a dual-source setup (^137^Cs and ^60^Co). The ^137^Cs source (activity 3.73 × 10^5^ Bq) was placed in a densely obstructed region at coordinates (4.4 m, 5.2 m), shielded by aluminum barrels. Its gamma-ray emissions were primarily directed along an azimuth of 180°. In contrast, the ^60^Co source (activity 3.94 × 10^5^ Bq) was located in an unobstructed area at (1.2 m, 5.7 m), with no surrounding obstacles causing shielding. Due to spatial constraints from the obstacle layout, the robot was required to navigate around obstructions, leading to a loss of continuous radiation measurements in approximately 23.5% of the experimental area

**Figure 3:**
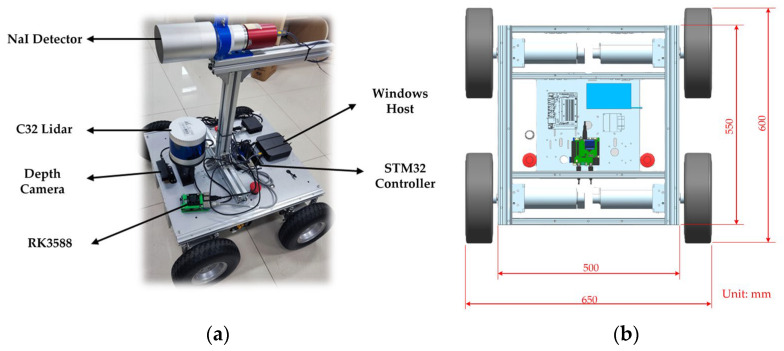
Radiation detection robot system architecture. (**a**) Physical view of the developed mobile robot equipped with NaI(Tl) detector, C32 LiDAR, depth camera, and RK3588 processor. (**b**) Mechanical layout of the robot chassis.

This experiment was conducted using a self-developed radiation detection robot system, whose hardware architecture is illustrated in Figure 3.

The data acquisition system consists of three core components: (1) RGB camera (ORBBEC, Shenzhen, China): mounted at the front of the robot, it provides visual feedback for remote operation and assists in preliminary data collection. (2) The C32 line laser radar (LiDAR) (LSLiDAR, Shenzhen, China): installed mid-front on the robot chassis, it offers 340° scanning coverage for obstacle detection and environmental mapping. (3) NaI(Tl) radiation detector (Jiechuang Nuclear Instrument, Beijing, China): positioned centrally on the robot, it performs real-time gamma radiation count measurements.

The software system is built on an embedded computing platform utilizing an RK3588 chip to handle high-level control and data processing. Chassis motion is managed via an STM32 controller. The robot operates on Ubuntu 20.04 with a Linux (Linux Ubuntu 20.04, Canonical Ltd., London, UK) kernel and utilizes the ROS framework with the Cartographer (Cartographer ROS (ROS1), Google (Opensource), Mountain View, CA, USA) package for real-time 2D SLAM. Radiation field mapping is visualized by ROS visualization (Rviz). Remote control is facilitated via NoMachine over a network connection. While the radiation levels in the experiment posed potential health risks to humans, they remained within safe limits for the robot’s internal electronic components. Therefore, no additional radiation shielding was applied to the electronic devices.

### 3.2. Collection of Sensor and Radiation Data

The effectiveness of the multi-kernel weighting method hinges on the kernel system’s ability to accurately model the physical mechanisms of radiation transmission. Consequently, the selection of kernel functions and their corresponding parameters must be predefined based on prior knowledge of the environment. To ensure physical interpretability, spatial topology information is first extracted through three-dimensional environment reconstruction. This enables the establishment of a mapping between material-specific attenuation characteristic and kernel parameters, thereby enhancing both the accuracy and robustness of the model.

**Figure 4:**
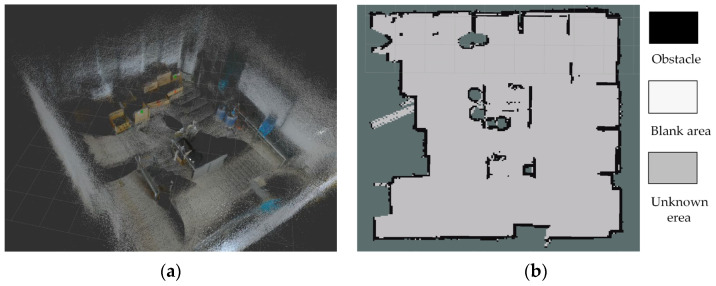
(**a**) The 3D reconstruction of the experimental environment; (**b**) 2D raster map of the experimental site. The map resolution is 0.05 m. Black represents obstacle regions, white indicates free space, and gray denotes unexplored or unknown areas.

Obstacle prior data acquisition: In this study, a self-development version of the keyframe-based ORB-SLAM3 algorithm [30] was employed to 3D point cloud reconstruction of the experiment layout. During data collection, a Gemini2 RGB-D camera (ORBBEC) served as the sensing unit, acquiring both depth and RGB images. These data were transmitted to the ORB-SLAM3 node for processing. After keyframe extraction and beam-based optimization, a three-dimensional point cloud model was generated, encoding the spatial structure and distribution of obstacles, as illustrated in Figure 4a.

Through point cloud registration, the geometric parameters and spatial distribution of obstacles were extracted from the reconstruction 3D model. This prior information served as a critical spatial constraint for design and weighting of the multi-kernel interpolation method. Specifically, kernel weights were assigned according to the spatial layout of obstacles, as detailed in Equation (8).

Environmental raster data acquisition: In parallel with point cloud reconstruction, 2D environmental mapping was conducted by using the Cartographer algorithm on a Linux system. With optimized parameters (submap size 8 m × 8 m, loop closure detection threshold 0.15), a 2D occupancy grid map of the environment was generated, providing a complementary spatial representation for radiation distribution reconstruction. The result map is shown in Figure 4b.

The generated raster map clearly delineates terrain features and obstacle contours, providing a reliable spatial reference for the fusion of radiation counts measurements.

Radiation data acquisition:

To obtain the spatial characteristics of radiation distribution, a non-uniform spatial sampling strategy was employed. Experiments were conducted separately under single-source (^137^Cs) and dual-source (^137^Cs and ^60^Co) scenarios, with approximately 200 measurement points collected per run. Gamma radiation counts were measured by the NaI(Tl), and data were logged as timestamped text files.

During robot operation, the count rate (gamma events per second, cps) was used as a relative measure of radiation intensity. Observed values included: background radiation ≈ 530 cps; peak near the ^137^Cs: 2313 cps; and peak near the ^60^Co source: 9831 cps.

After data acquisition, radiation counts data and corresponding timestamps were transmitted to the RK3588 platform via PTP (Precision Time Protocol). Subsequent data synchronization with LiDAR scans and spatial mapping were performed within the Linux operating environment, enabling accurate fusion of spatial and radiation data.

### 3.3. Construction and Analysis of the Radiation Field Map

**Figure 5:**
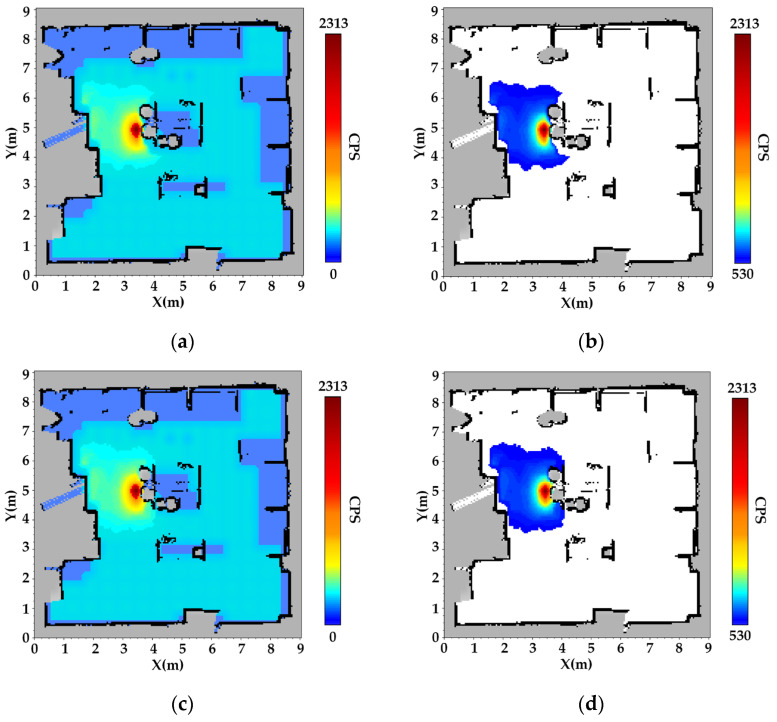
Radiation field reconstruction results for the ^137^Cs single-source scenario. (**a**) Global radiation distribution using the MK-GPR model. This map highlights anisotropic attenuation effects around the source due to obstacle shielding, with radiation patterns deviating from radial symmetry. (**b**) Local radiation field (after background removal) using MK-GPR. The source hotspot and its radiation shadow caused by the aluminum barrel (diameter 0.4 m) are clearly visible. (**c**) Global radiation distribution using a single-kernel GPR model. The interpolation exhibits a symmetric and circular radiation field, disregarding the impact of obstacles and resulting in an unrealistic spread. (**d**) Local radiation field (after background removal) using single-kernel GPR. No significant attenuation is observed across obstacle regions, inconsistent with physical expectations of γ-ray behavior.

**Figure 6:**
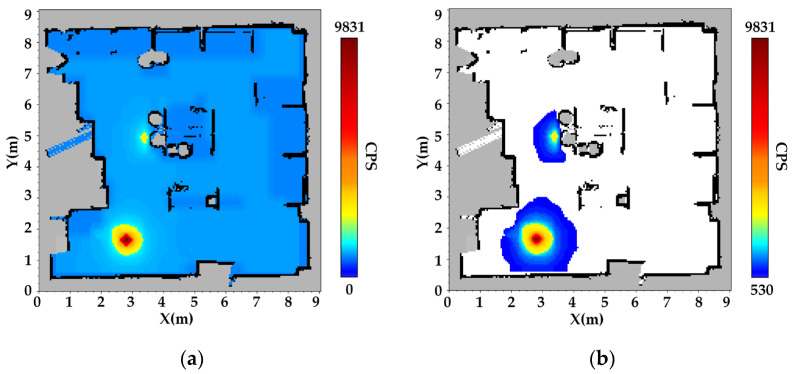
Radiation field map for ^137^Cs and ^60^Co sources using the MK-GPR method. (**a**) Global radiation intensity distribution: This map illustrates the overall radiation field resulting from a weaker ^137^Cs source in the upper region and a stronger ^60^Co source in the lower region. (**b**) Local radiation field around the ^60^Co source after background removal: This map highlights the radiation intensity distribution after eliminating the background contribution, revealing detailed field gradients in the vicinity of the ^60^Co source.

Following data acquisition, the radiation counts distribution map was constructed using a multi-kernel weighted interpolation method. Spatiotemporal data registration across multiple sensor modalities was implemented within the ROS framework, enabling precise alignment of radiation counts with SLAM-derived coordinates via timestamp synchronization. The radiation counts presented in Figure 5 and Figure 6 represent the total gamma-ray count rates (cps) measured by the NaI(Tl) detector. These counts reflect the combined contributions of the ^137^Cs and ^60^Co sources in the dual-source scenario, as well as background and scattered radiation. The interpolated radiation mapping for single-source scenario ^137^Cs is presented in Figure 5, based on the generated occupancy grid map and the fused spatial data.

In the constructed radiation map, gray regions represent unknown areas—either occluded by obstacles or corresponding to their interiors. Black regions denote obstacle boundaries and terrain features, consistent with SLAM-derived structural mapping. Color gradients ranging from blue to yellow to red indicate increasing radiation counts, with color saturation positively correlated with radiation levels.

The results indicate that the spatial deviation between the radiation hotspot (dark red area) and the actual location of the ^137^Cs source (4.4 m, 5.2 m) is only 0.1 m, demonstrating high spatial accuracy. Furthermore, the discrepancy between the radiation shadow region (gray circular area) and the actual aluminum barrel (diameter 0.4 m) is less than 5%. As shown in Figure 5b, radiation distribution exhibits exponential attenuation in the vicinity of obstacles, confirming that the Gaussian multi-kernel weighted model effectively captures the shielding effects induced by barriers.

Figure 5c, d presents the radiation field reconstructions using a single-kernel Gaussian process regression (GPR) approach. This baseline model utilizes a modified inverse-square (InvSq) kernel, designed to reflect the free-space attenuation pattern of γ-rays. However, it does not incorporate environmental structures or obstacle information during interpolation, resulting in oversimplified and physically inconsistent reconstructions in obstructed regions.

Unlike the MK-GPR results shown where obstacle-induced shielding leads to a fan-shaped radiation pattern, the single-kernel model fails to capture any significant directional attenuation. In particular, radiation intensity appears nearly uniform before and after penetrating the obstacle, contradicting the physical reality of γ-ray attenuation in high-density materials. This further demonstrates the necessity of incorporating spatial priors and obstacle geometry in radiation mapping to achieve physically consistent interpolation results. The results indicate that the spatial deviation between the reconstructed radiation hotspot and the actual location of the ^137^Cs source is approximately 0.3 m.

In the dual-source scenario, the reconstructed radiation map for both ^137^Cs and ^60^Co sources is shown in Figure 6.

The dual-source experimental scenario involves two gamma radiation sources with differing intensities: a weaker ^137^Cs source positioned in the upper region and a stronger ^60^Co source located in the lower region. As expected, the ^137^Cs source produces significantly lower radiation counts compared to the ^60^Co source. In the overlapping region influenced by both sources, a moderate increase in cumulative radiation counts is observed, which reflects the superposition of radiation fields.

The experimental results demonstrate that the MK-GPR method can accurately and independently reconstruct radiation distributions from multiple sources, even under complex conditions involving shielding and source overlap. Specifically, the radiation pattern of the ^137^Cs source, which is partially shielded by physical obstacles, exhibits a dual-attenuation behavior: exponential decay due to material attenuation and inverse-square reduction with distance from the source. In contrast, the ^60^Co source, situated in a relatively unobstructed area, generates a radiation field that closely adheres to the theoretical inverse-square law, with counts diminishing radially from the source center. The spatial gradients of the measured radiation counts show strong agreement with theoretical physical models, further validating the fidelity and physical consistency of the reconstructed radiation fields. This indicates that the MK-GPR method effectively captures both gradual and abrupt spatial transitions in radiation intensity, depending on environmental features.

It is important to note that the attenuation model employed idealized material parameters based on pure metals such as aluminum and iron. However, some obstacles in the test environment were composed of composite materials with mixed or unknown attenuation characteristics, although these materials still exhibited exponential attenuation for gamma rays. This material mismatch may have introduced minor deviations in the estimated attenuation coefficients. To evaluate the sensitivity of the MK-GPR method to uncertainties in material parameters, calculations were performed using the NIST XCOM database to simulate the impact of material composition variations on the predicted radiation intensity. For the aluminum obstacle adjacent to the ^137^Cs source, the aluminum mass fraction was reduced from 100% to 75%, and the iron content was set to 25%, resulting in linear attenuation coefficients (μ) for 662 keV gamma rays ranging from 20.16 m^−1^ to 29.58 m^−1^. The reconstructed radiation field indicates that a 25% change in aluminum content leads to a maximum deviation of approximately 6% in the predicted radiation intensity in obstacle-shadowed regions. This demonstrates that while the MK-GPR method exhibits strong robustness to moderate material uncertainties, precise material characterization remains essential for achieving high-accuracy radiation mapping. A quantitative comparison of source localization performance is provided in Table 3, further supporting the robustness of the proposed method in complex, multi-source scenarios.

It should be noted that these results were obtained in a controlled laboratory environment with predefined source configurations (single and dual sources at fixed positions). Real-world environments involve more factors to consider and are significantly more complex than laboratory settings.

Experimental results demonstrate the efficiency of the MK-GPR method for high-precision radiation mapping. In single-source scenarios, localization errors were constrained within 0.10 m. For the dual-source case, the average localization error remained below 0.15 m. Moreover, the method illustrated strong adherence to physical change law via distance and material attenuation.

**Figure 7:**
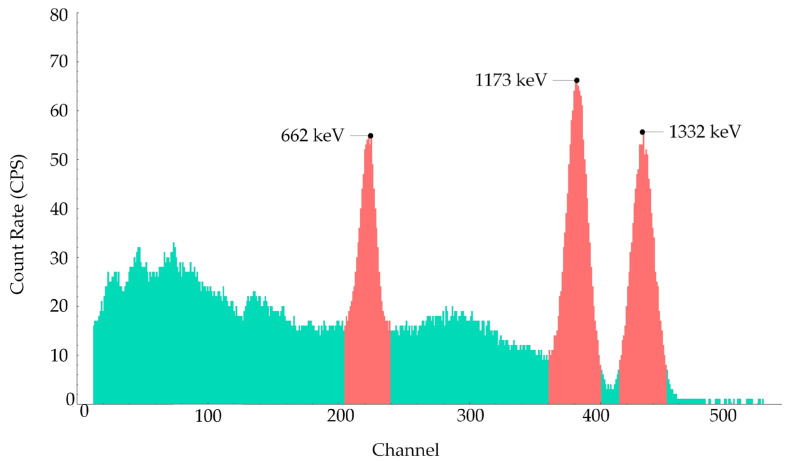
Gamma-ray energy spectrum acquired by the NaI(Tl) detector in a mixed-source environment. The turquoiseregion represents background and scattered radiation; the red peaks correspond to characteristic photo peaks of ^137^Cs (662 keV) and ^60^Co (1173 and 1332 keV). The energy channels 223, 382, and 433 correspond to the energies 662, 1173, and 1332 keV, respectively, reflecting the calibration of the energy spectrum.

Following source localization, gamma-ray spectra analysis was performed for radiation characterization. As illustrated in Figure 7, for the dual-source scenario, distinct photo peaks corresponding to specific isotopes were observed: 662 keV from ^137^Cs, 1173 keV, and 1332 keV from ^60^Co. This spectra analysis capability augments the radiation mapping system’s overall effectiveness by enabling isotopic identification.

In multi-source scenarios, if it is necessary to assess the contribution of each radiation source to the overall intensity, Gaussian process regression can be performed separately for each source based on the characteristic peak counts extracted from Figure 7. This approach enables source separation and spatial reconstruction of individual source distributions.

In conclusion, the MK-GPR method effectively mitigates the limitations of conventional models in accounting for obstacle-induced shielding effects during radiation mapping. This work provides a robust technical basis for enhanced radiation monitoring and emergency response operations within complex environments.

### 3.4. Discussion

The MK-GPR method’s ability to model complex attenuation patterns in occluded environments enhances its utility in nuclear safety applications. For instance, in decommissioning tasks at sites like Fukushima Daiichi, MK-GPR can map radioactive hotspots amidst heterogeneous debris, as demonstrated by its robustness to material uncertainties. It also supports rapid source localization in contaminated indoor settings and radioactive debris mapping, improving emergency response and cleanup efficiency.

Despite its overall strong performance, MK-GPR still has certain limitations. First, there are material property mismatches. Applying attenuation coefficients of ideal metals (e.g., aluminum and iron) to composite or unknown materials may introduce systematic errors. To address this limitation, future work will integrate on-site material characterization techniques, such as X-ray fluorescence or neutron activation analysis, to determine the composition of composite materials. Second, temporal misalignment during the robot’s movement may lead to slight registration errors between radiation counts and spatial positions. Addressing these issues could further improve the accuracy and robustness of the method in real-world deployments.

While the current MK-GPR framework is designed and validated for gamma-ray fields only, future work could explore its extension to mixed radiation fields by integrating data acquired from liquid scintillator detectors, which are sensitive to both gamma rays and neutrons. Such an extension would improve the applicability of the method in post-accidental or mixed-field radiation environments.

Although the proposed MK-GPR method was validated in a controlled 8 m × 8 m indoor environment, it is inherently scalable in both algorithmic design and system deployment. This enables its application in larger, more complex scenarios, such as cluttered industrial sites or damaged nuclear facilities. In our experiments, approximately 200 radiation measurements were collected, with the full data acquisition and reconstruction process completed in about 8 min. The RK3588 embedded platform maintained an average computational load of 4.13. Notably, the reconstruction accuracy depends more on the spatial distribution of measurement points than on their total number, allowing high-fidelity results without requiring dense sampling.

When extended to environments on the order of 50 m × 50 m or more, increases in data volume and computational cost remain manageable within the platform’s capabilities. Moreover, the spatial resolution of the radiation map is influenced by factors such as grid point density, interpolation strategy (e.g., diagonal schemes), the nonlinear relationship between resolution and sample count, and the precision of radiation measurements. Addressing these aspects holistically will further support the deployment of MK-GPR in real-world, high-resolution radiation mapping tasks.

## 4. Conclusions

In response to the essential challenge of rapidly and precisely investigating radiation sources after accidents or disasters, which often involve structural damage and radiation shielding by obstacles, this study proposed a multi-kernel Gaussian process regression (MK-GPR) model for high-precision radiation mapping. Based on the traditional single Gaussian kernel that models free-space inverse-square law decay, the MK-GPR innovatively combines kernels that represent the attenuation characteristics of obstacle materials and the energy-dependent penetration capabilities of radiation through a weighted formulation. This enables the model to accurately reflect the abrupt radiation field gradients caused by obstacles.

Regression experiments using radiation distribution data simulated in obstructed spaces with Geant4 demonstrated that MK-GPR outperforms traditional single-kernel GPR. Specifically, MK-GPR achieved a 28% reduction in root mean squared error (*RMSE*), a 26% reduction in mean absolute error (*MAE*), and a 48% decrease in mean squared error (*MSE*). Additionally, the coefficient of determination (*R*^2^) significantly improved to 0.937, confirming MK-GPR’s effectiveness in capturing data to accurately characterize radiation gradient changes at obstacle boundaries.

To advance its practical application, the optimized MK-GPR algorithm was deployed on an RK-3588 edge computing device and integrated into a mobile robot platform equipped with a NaI(Tl) detector. Combined with Simultaneous Localization and Mapping (SLAM) technology, this system successfully performed radiation field mapping in a simulated obstructed laboratory environment. This setup achieved precise localization of radiation sources on a grid map, with localization errors of 10 cm for single sources and 15 cm for dual sources.

The radiation-geometric grid maps generated by the MK-GPR method provide field personnel with an intuitive and accurate reference for radiation distribution. This offers a deployable and practical solution for the rapid and precise search and localization of radiation sources in post-disaster environments, exhibiting broad application prospects in nuclear emergency response and routine inspections.

Future research will focus on exploring the integration of MK-GPR with path planning techniques and adaptations for non-point source scenarios, such as surface contamination, through enhanced detector configurations. This aims to optimize the robot’s autonomous detection and navigation capabilities in complex obstructed environments, providing more intelligent and efficient solutions for radiation safety.

## Figures and Tables

**Table 1:** Linear attenuation coefficients for aluminum and iron under different gamma-ray energies.

Material	^137^Cs (662 keV)	^60^Co (1173/1332 keV)
Aluminum (Al)	20.16 ($\mu_{Cs}^{Al}$) *	14.85 ($\mu_{Co}^{Al}$)
Iron (Fe)	57.84 ($\mu_{Cs}^{Fe}$)	42.16 ($\mu_{Co}^{Fe}$)

* The values represent the linear attenuation coefficients ($\mu_{E}^{m}$, in $m^{-1}$) for aluminum (Al) and iron (Fe) at different gamma-ray energies. Specifically, 20.16 and 14.85 correspond to Al at 662 keV (^137^Cs) and 1173/1332 keV (^60^Co), respectively; 57.84 and 42.16 correspond to Fe at the same two energies.

**Table 2:** Error Analysis and computational performance comparison of different interpolation methods.

	*RMSE*	*MSE*	*MAE*	*R* ^2^	Comp Time (s)	Comp Load
Linear Interpolation	8.65	74.81	6.98	0.868	0.36	1.42
Nearest Interpolation	8.42	70.98	5.58	0.874	0.56	1.64
Gaussian Single-Kernel	8.28	68.54	6.22	0.879	0.97	3.97
Gaussian Multi-Kernel	5.97	35.63	4.61	0.937	1.15	4.13

**Table 3:** Radiation source coordinates and reconstructed positions.

	Single Radioactive Source	Dual Radioactive Source	
Radiation Source	^137^Cs	^137^Cs	^60^Co
Activity (Bq)	3.73 × 10^5^	3.73 × 10^5^	3.94 × 10^5^
Actual Position (m)	(4.40, 5.20)	(4.40, 5.20)	(1.20, 5.70)
Grid Coordinates	(68, 99)	(68, 100)	(58, 35)
Reconstructed Coordinates (m)	(4.40, 5.30)	(4.45, 5.30)	(1.30, 5.80)
Positioning Error (m)	0.10	0.12	0.14

## Data Availability

Data are contained within the article.
